# Sigmoid colonic metastasis from a squamous cell carcinoma of the cervix: A rare case report with literature review

**DOI:** 10.1097/MD.0000000000037001

**Published:** 2024-01-19

**Authors:** Minhua Li, Weiping Zheng

**Affiliations:** a Departments of Pathology, Shaoxing People’s Hospital, Shaoxing, Zhejiang, China.

**Keywords:** cervical cancer, gastrointestinal tract, intestinal obstruction, sigmoid colonic metastasis, squamous cell carcinoma

## Abstract

**Rationale:**

As the third most common cancer in women, cervical cancer usually spreads to adjacent organs. Distant metastasis from the cervix to the gastrointestinal tract is an extremely rare occurrence.

**Patient concerns:**

Herein, we present a rare case of a 57-year-old woman who was treated by hysterectomy and bilateral salpingo-oophorectomy with pelvic lymphadenectomy for squamous cell carcinoma (SCC) of the uterine cervix. A metastatic location in the sigmoid colon was revealed after 8 years causing an acute intestinal obstruction in this patient.

**Diagnoses:**

Final surgical pathology showed an invasive lesion with squamous differentiation in full thickness of the colon wall from mucosa to serosa. Meanwhile, the results of immunohistochemistry (IHC) showed the cancer cells were positive for CK5/6, P63, P40, and P16 confirming the diagnosis of metastatic sigmoid colonic carcinoma originating from SCC of the uterine cervix.

**Interventions:**

Sigmoid colon resection with lymph node dissection followed by adjuvant chemotherapy (paclitaxel, carboplatin, and paprillizumab) was performed on the patient.

**Outcomes:**

The patient was disease-free 16 months after surgery.

**Lessons subsections:**

SCC is one of the rare malignant tumors of the gastrointestinal tract occurring as either a primary or secondary lesion. However, the secondary SCC of the colon has a poorer prognosis compared with the primary SCC. Therefore, colonic metastasis must be considered in the differential diagnosis of acute intestinal obstruction, especially in patients with the medical history of SCC in other organs.

## 1. Introduction

Cervical cancer is one of the worldwide malignant disease, ranking the third most common cause of cancer death among women.^[1]^Histologically, approximately 70% of all cervical cancers were squamous cell carcinoma (SCC) and the incidence of human papillomavirus (HPV) infection accounted for 90% of all cervical SCC.^[2]^ Metastasis or recurrence was usually associated with an overall poor prognosis in the patient of cervical SCC. Lungs or paraaortic nodes were the most common sites in distant metastasis. Occasionally, bones, liver, spleen, muscle, skin and brain were included as well. Gastrointestinal metastasis from the cervical SCC is an extremely rare occurrence and only a handful of case reports were described until now. In the past 3 decades, only 10 cases of metastatic carcinoma from the cervix to the colon have been published in the literature to date^[3–12]^ (Table 1). Physicians should be pay more attention this rare metastatic locations of cervical SCC to the sigmoid colon for appropriate diagnosis and treatment of these patients.

**Table 1:** Colonic metastases from cervical cancer as reported in the literature.

NO	Reference	Age	Stage of SCCA	Previous treatment	Interval time	Symptom	Metastasis sites	Treatment	Outcome
1	Joshi, S. R.^[3]^	50	Stage II	Wertheim hysterectomy	5 mo	Abdominal pain, vomiting , intermittent fever	Ileocaecal region	Segmentary intestinal resection	NA
2	Datta, S.^[4]^	55	Stage IIB	Chemoradiation	3.5 yr	Abdominal pain, vomiting, constipation	Ileocaecal region	Right hemicolectomy	NA
3	Hui Qiu^[5]^	46	Stage IIB	Chemoradiotherapy	4 yr	Acute abdominal pain	Ileocaecal region	Segmentary intestinal Resection	Recovery for 2 yr
4	Rockson O.^[6]^	59	Stage IIB	Wertheim hysterectomy and adjuvant chemoradiation	2 yr	Acute intestinal obstruction, abdominal pain, vomiting	Cecum	Right hemicolectomy	Disease-free 11 mo after surgery
5	Dey A.^[7]^	56	Stage II	hysterectomy followed by radiation therapy	14 mo	Abdominal wall abscess	Ascending flexure of the colon	Right hemicolectomy	Disease free 2 mo after surgery
6	Singla, M.^[8]^	48	NA	Radiation therapy	2 yr	Right hypochondrium pain	Hepatic flexure of colon	Right extended hemicolectomy	Recovery for 2 yr
7	Nawarathna N. J.^[9]^	62	Stage III	Wertheim hysterectomy, adjuvant radiotherapy	3 yr	Acute intestinal obstruction	Transverse colon	Segmentary intestinal resection	Disease-free
8	Barlin, J. N^[10]^	37	Stage IB	Radical hysterectomy	1.5 yr	Hematochezia	Sigmoid colon	Rectosigmoid resection	Recovery
9	Yu X.^[11]^	45	IB1	Hysterectomy with adnexectomy.	3 yr	Abdominal distention and dull pain, vomiting	Sigmoid colon and small intestine	Segmental intestine resection	Disease free 4 mo after surgery
10	Lelchuk A.^[12]^	47	NA	Hysterectomy	4 yr	Large bowel obstruction	Sigmoid colon	Rectosigmoid colon resection	NA
11	Present case	57	IB1	Hysterectomy with adnexectomy.	8 yr	Acute intestinal obstruction, abdominal pain	Sigmoid colon	Sigmoid colon resection	Disease-free 16 mo after surgery

In this report, we describe a rare case of a 57-year-old woman with a history of cervical SCC, metastasizing to the sigmoid colon 8 years after a hysterectomy and bilateral salpingo-oophorectomy treatments with pelvic lymphadenectomy was performed. Moreover, the clinicopathological features, management strategies, and the prognosis of this rare entity were discussed by reviewing all of the literature.

## 2. Case presentation

A 57-year-old Chinese female was admitted to the emergency unit of our hospital with a chief complaint of episodic abdominal pains without passage of stools or flatus for 4 days. At physical examination, all the vital signs were stable in this patient. Abdominal tenderness and rebound tenderness was unremarkable, however, abdominal light distension and a loud gurgling sound was found. Digital rectal examination did not revealed any mass at the lower part of the rectum and bimanual vaginal examination did not showed any obvious abnormalities. Laboratory tests showed the tumor markers were normal and the analysis of blood tests was similar to the normal baseline. Meanwhile, no lesions from esophagus, oropharynx, nasopharynx, and lung were founded.

**Figure 1.**
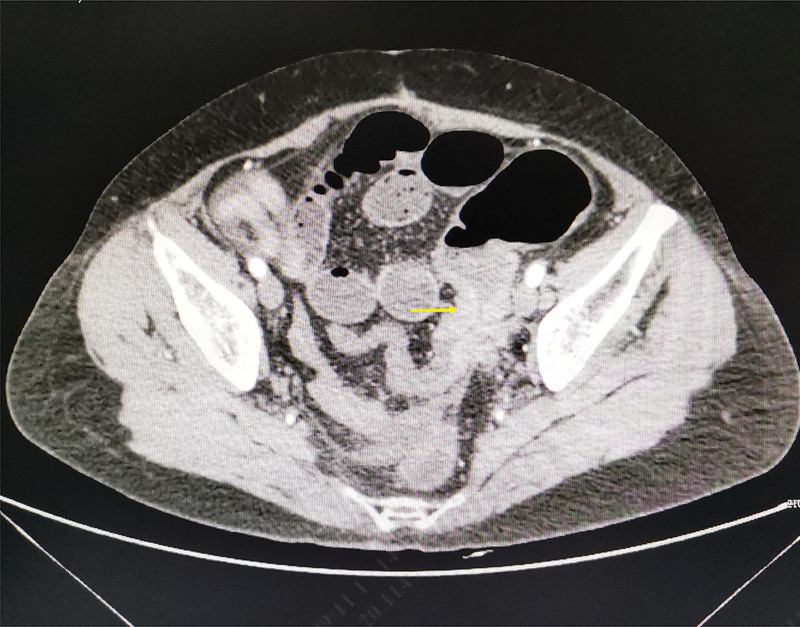
Contrast-enhanced CT image shows a mass, narrowing the lumen of the sigmoid colon. CT = computed tomography.

At the same time, we take a careful medical history. Eight years prior to the current presentation, she had radical hysterectomy and adnexectomy with pelvic lymphadenectomy for cervical cacinoma in outside hospital. The postoperative pathological examination showed a moderately differentiated squamous cell carcinoma in uterine cervix, measuring 35*17 mm with infiltration depth to 8 mm. All thirty-six pelvic lymph nodes were free of any carcinoma. According to the International Federation of Gynaecology and Obstetrics classification, it was diagnosed with stage IB1 SCC of the cervix. For further exploring the cause of abdominal pains, abdominal conventional computed tomography (CT) scan was performed, revealing a thickened bowel wall in the sigmoid colon with lower intestinal obstruction. Then, contrast-enhanced CT image shows huge mass in the sigmoid colon (Fig. 1), which was accompanied with multiple lymph node enlargement, and the lower end of ureters was involved by the tumor, resulting in hydronephrotic changes. Therefore, radical sigmoid colon resection with lymph node dissection was performed.

The tissue was fixed in formalin and embedded in paraffin, after which 4 µm thin sections were cut and stained with hematoxylin and eosin. Immunohistochemical staining was performed using commercially available antibodies to the following antigens: CKpan, CK7, CK20,CDX2, P63, P40, CK5/6, P16, MLH1, MSH2, MSH6, PMS2, and Ki-67.All protocols were employed according to the manufacturers’ recommendations (Table 2).

**Table 2:** Summary of primary antibodies and results of immunohistochemistry.

Antibody	Source	Dilution	Result
CK	Ascend Bio, Guangzhou, China	1:100	+
CK20	Ascend bio, Guangzhou, China	1:200	-
CK7	Ascend bio, Guangzhou, China	1:100	-
CDX2	Ascend bio, Guangzhou, China	1:400	-
CK5/6	ZSGB bio, Beijing, China	1:1600	+
P63	ZSGB bio, Beijing, China	1:400	+
P40	ZSGB bio, Beijing, China	1:200	+
P16	ZSGB bio, Beijing, China	1:800	+
Ki-67	Dako, Glostrup, Denmark	RTU	80% in the most concentrated spot
MLH1	Dako, Glostrup, Denmark	RTU	-
MSH2	Dako, Glostrup, Denmark	RTU	-
MSH6	Dako, Glostrup, Denmark	RTU	-
PMS2	Dako, Glostrup, Denmark	RTU	-

CK = cytokeratin, RTU = ready to use.

**Figure 2.**
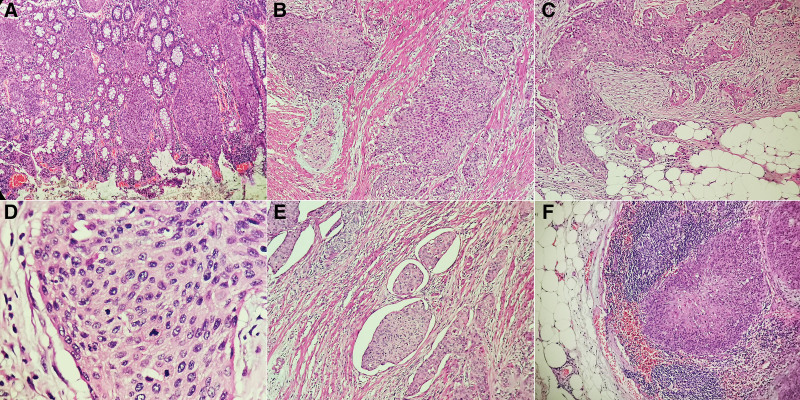
(A) Atypical neoplastic cells with nest structure was revealed in the mucosa of the sigmoid colonic wall (×200). (B) The muscular layer of sigmoid colon was destroyed by the carcinoma cells (×200). (C) Fibrous and fatty tissue was invaded by the carcinoma cells (×200). (D) Obvious abnormal mitoses and nuclear atypia with squamous features were frequently seen (×400). (E) The vessel invasion was revealed in some part of the lesion (×200). (F) Metastatic carcinoma was present in lymph node (×200).

**Figure 3.**
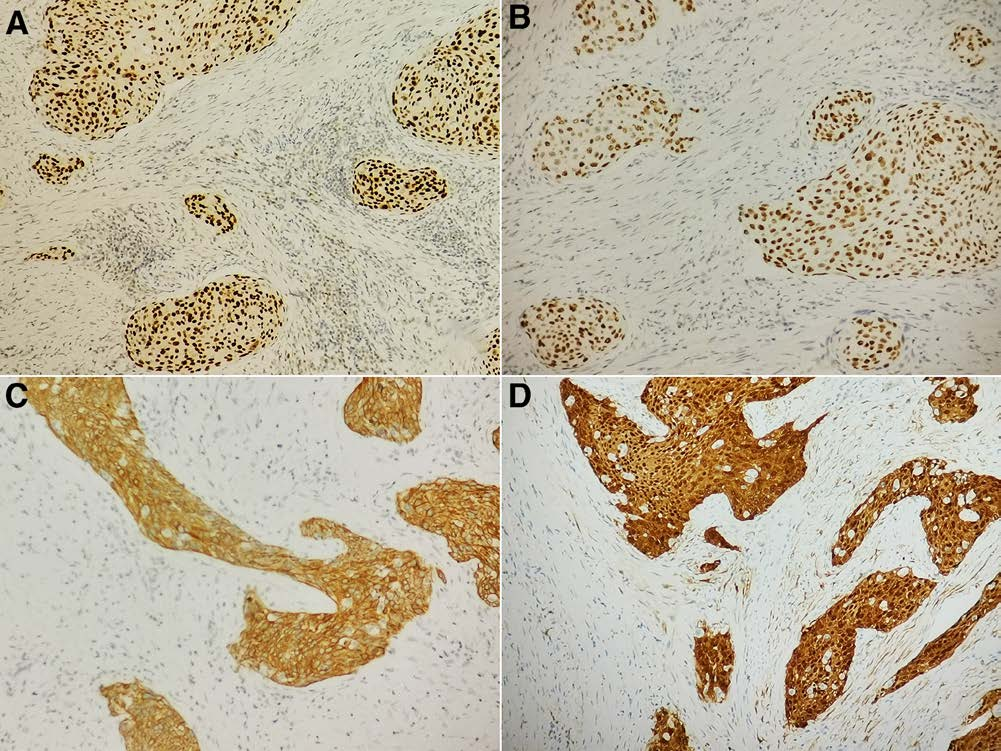
(A) Definitely positive staining of P63 was present in tumor cells (×200). (B) Positive expression of P40 was evident (×200). (C) Strong staining of CK5/6 was displayed (×200). (D) Strong positive expression of P16 was revealed (×200).

Macroscopically, an ulcerated mass with irregular elevations at the margins accounted for all of the circumference of narrow lumen in the middle part of sigmoid colon, measuring 50*35 mm. Final histological examination of the neoplasm revealed an invasive lesion with squamous features in full thickness of the colon wall from mucosa to serosa (Fig. 2A–C). The tumor was composed of large numbers of atypical neoplastic cells with nest structure. Obvious abnormal mitoses and nuclear atypia with squamous features were frequently seen (Fig. 2D). Moreover, the vessel invasion was revealed in some part of the lesion and metastatic carcinoma was present in twelve of twenty-six lymph nodes (Fig. 2E,F).The results of IHC showed the cancer cells were positive for CKpan, P63, P40, CK5/6, and P16 (Fig. 3A–D), but negative for CK7, CK20, CDX2, MLH1, MSH2, MSH6, and PMS2.The Ki67-labeling index reached 80% in the most concentrated spot. Therefore, we made a diagnosis of metastatic sigmoid colonic carcinoma originating from SCC of the uterine cervix in this patient.

Until now, she is disease-free 16 months after sigmoid colon resection with lymph node dissection followed by adjuvant chemotherapy(paclitaxel, carboplatin and paprillizumab) was performed.

## 3. Discussion

With the development of screening and advances in chemoradiotherapy, the survival rates of cervical cancer have greatly improved. However, the rates of recurrence and metastasis in the patients of cervical cancer has also been increased, which play an important role in the main causes of death.^[11]^ The most common sites were lungs or paraaortic nodes in distant metastasis of cervical cancer. Gastrointestinal metastasis from the cervical cancer is extremely rare.

In the past 3 decades, only 10 cases of metastatic carcinoma from the cervix to the colon have been documented in the literature.^[3–12]^ Clinicopathological features of the published cases were listed in Table 1. The median age is 51 years in the patients, which ranged from 37 to 62 years. The average interval time from discovering the primary cancers to metastasis was 30 months, which ranged from 5 months to 4 years. In our case, the interval time was 8 years, showing the longest time among the all the cases. The most of metastasis located in the cecum and right colon, merely 3 cases occurring in sigmoid colon. To date, our case report was the fourth case of cervical SCC metastasizing to the sigmoid colon. Acute abdominal pain, vomiting and intestinal obstruction were the main clinical symptom, occasionally, abdominal wall abscess or hematochezia were present in the patients. Acute intestinal obstruction and abdominal pain were present in our case. An advanced tumor stage was revealed in the majority of the cases and most of them were SCC, only one case showed adenosquamous carcinoma, which was consistent with our case. After surgery, the patient could acquired disease-free from 2 months to 2 years. The patient in our case showed disease-free 16 months until now. Further follow-up was needed in our case for investigating this rare cervical SCC metastasizing to the sigmoid colon.

Most of the colonic carcinomas are adenocarcinomas due to the glandular epithelium of colorectal mucosa. The other carcinomas only account for 10% of colonic carcinomas, including SCC, undifferentiated carcinomas, neuroendocrine tumors, lymphomas, and so on. SCC is one of the rare malignant tumor of the gastrointestinal tract occurring as either a primary or secondary lesion. Colonic metastasis is known to arise from primary sites such as the breast, kidney, ovary.^[12]^ At the same time, due to the relatively short intestinal segment, sigmoid colon metastases are extremely rare. Because the secondary SCC of the colon has a poorer prognosis compared with primary SCC, it is important to differentiate between these 2 entities.

In 1979, 3 diagnostic criteria was proposed to help distinguish primary SCC from metastatic SCC:the lack of existence of a non-colonic primary SCC; exclusion of SCC of the anus with proximal extension; the presence of squamous-epithelial lined fistula to the tumor site must be eliminated.^[13]^ In our case, these criteria were not present in the patient, so it was impossible to diagnosis the primary squamous cell carcinoma. Taken into account the medical history of SCC in the cervix and exclusion of possible primary lesions from esophagus, oropharynx, nasopharynx, and lung cancer, it is most likely that sigmoid colonic tumor was a metastasis of cervical cancer. Moreover, the results of IHC showed the cancer cells were positive for P63, P40, and CK5/6,further hinting the origination of squamous cell epithelium differentiation. At the same time, as a surrogate marker for high-risk HPV infection, P16 associated extremely well with high-risk HPV status. In our case, strong positive expression of P16 was also revealed, further confirming the diagnosis of metastatic sigmoid colonic carcinoma originating from SCC of the uterine cervix.

Secondary tumor of colon frequently result from metastatic disease process, peritoneal seeding or direct spread. It has been proposed that hematogenous or lymphatic dissemination may play an important role in metastatic disease process. In our case, the existence of vessel invasion was founded, so hematogenous spread is most likely. However, possibility of retrograde lymphatic permeation could not be completely excluded due to the presence of metastatic carcinoma in twelve of twenty-six lymph nodes, which surrounded the sigmoid colon.

As for the optimal treatment for secondary SCC in sigmoid colon, no consensus was reached until now. Generally, the primary treatment modalities were surgical resection and debulking of the carcinoma. Whether the radiotherapy and chemotherapy play an important role in clinical therapy remain somewhat controversial. Maybe chemoradiotherapy could be employed as a palliative measures.^[12]^ Because the lack of enough available data to compare the efficacy of the different measures, further investigation is needed in the future.

## 4. Conclusions

In a conclusion, we present a rare case report of SCC of the cervix metastasizing to the sigmoid colon presenting as an acute intestinal obstruction. Though there was existence of primary SCC in sigmoid colon, metastasis must be considered in the differential diagnosis of acute intestinal obstruction, especially in patients with medical history of SCC in other organ. Moreover, due to no specific symptoms and late presentation, most of sigmoid colon metastasis acquired a poor prognosis^11^. Early detection and prompt intervention play an important role in saving the patients from possible fetal complications, such as intestinal perforation. Therefore, clinicians should pay more and more attention on this rare secondary carcinoma for achieving good palliation in the patients.

## Author contributions

**Investigation:** Weiping Zheng.

**Project administration:** Minhua Li.

**Writing – original draft:** Weiping Zheng.

**Writing – review & editing:** Minhua Li.
